# Device-measured and self-reported physical activity during the first two years postpartum in women with recent gestational diabetes: evidence from the LINDA-Brasil study

**DOI:** 10.20945/2359-4292-2024-0479

**Published:** 2025-07-22

**Authors:** Gabriela Feiden, Danilo de Paula, Natan Feter, Leony Galliano, Paula Bracco, Maria Inês Schmidt

**Affiliations:** 1 Programa de Pós-graduação em Epidemiologia, Universidade Federal do Rio Grande do Sul, Porto Alegre, RS, Brasil; 2 Programa de Pós-graduação em Educação Física, Universidade Federal do Rio Grande do Norte, Natal, RN, Brasil

**Keywords:** Gestational diabetes mellitus, postpartum, physical activity

## Abstract

**Objective:**

To quantify moderate-to-vigorous physical activity (MVPA) at postpartum in women with recent gestational diabetes mellitus, using an accelerometer and self-reported measurements from participants of the LINDA-Brasil study.

**Materials and methods:**

In a cross-sectional sample (n = 391), MVPA was assessed via a waist-worn accelerometer and the international physical activity questionnaire (IPAQ), focusing on leisure time and commuting domains.

**Results:**

The median postpartum period was 7.3 months (interquartile range [IQR]: 4.0-14.0). When restricted to 10-minute bouts, device-measured MVPA was 22.31 minutes/week (IQR: 0-65.8), whereas total time spent on MVPA was 213.8 minutes/week (IQR: 137.7-320.0). Higher education and pregnancy complications were associated with lower device-based MVPA. Self-reported leisure-time MVPA in 10-minute bouts was 0 minutes/week (IQR: 0-0). However, including commuting time, it increased to 90 minutes/week (IQR: 10.0-210.0). Based on total device-measured MVPA, 71.6% (CI 66.9-76.0) met the recommended 150 minutes/week. This proportion decreased to 8.4% (95% CI: 5.9-11.7) in 10-minute bouts MVPA. Based on the IPAQ, 7.4% (95% CI: 5.0-10.5) reached the guideline through leisure-time activity and 26.8% (95% CI: 22.5-31.5) through combined leisure and commuting.

**Conclusion:**

Women with gestational diabetes mellitus at postpartum were highly active based on device-measured MVPA. Nevertheless, applying the 10-minute bout reduced these estimates across devices and self-reported measurements. These findings provide crucial information for public policies addressing this high-risk population.

## INTRODUCTION

The global rise of type 2 diabetes parallels increasing obesity rates (^1^). Gestational diabetes mellitus (GDM), the strongest predictor of diabetes type 2 in women (^2^), may be mitigated through postpartum lifestyle interventions, especially physical activity (^3^-^5^). Although the World Health Organization (WHO) recommends at least 150 minutes of moderate/vigorous physical activity (MVPA) per week for all women during pregnancy and postpartum (^6^), few studies have assessed postpartum physical activity levels , particularly using device-measured physical activity in women with recent GDM (^7^-^11^).

Assessing physical activity (PA) levels at postpartum in women with recent GDM is essential for managing long-term metabolic disease and preventing future chronic conditions (^12^). Questionnaires can offer valuable contextual information, such as PA across leisure time, travel, household, or work activity. Self-reported leisure-time PA decreased from preconception to 12 months postpartum and remained lower at 48 months postpartum despite subsequent increases (^13^). Moreover, higher educational levels and income were associated with an increased likelihood of inactivity during pregnancy and postpartum (^13^).

Device-measured PA addresses key limitations of questionnaires, including recall and social desirability biases (^12^,^13^). However, implementing it requires a complex logistic organization, extensive financial resources, and specialized data processing and interpretation expertise. As a result, studies among women with recent GDM at postpartum remain scarce and confined to high-resource settings (^7^-^10^). To address this gap, we evaluated participants of the LINDA-Brasil study, which comprises women with recent GDM residing in six cities in Brazil. Thus, this study describes the time spent in MVPA based on both accelerometer and self-reported measures and its association with sociodemographic and perinatal characteristics.

## MATERIALS AND METHODS

### Study design and sample

This cross-sectional analysis used baseline information from the LINDA-Brasil study, conducted between 2015-2020, across six Brazilian cities: Porto Alegre, Pelotas, Fortaleza, São Paulo, Rio de Janeiro, and Curitiba. Eligible participants were women aged ≥18 years with a diagnosis of GDM in their most recent pregnancy within the past two years, who either received medication for GDM or experienced postpartum hyperglycemia not meeting diabetes criteria. All participants underwent an oral glucose tolerance test postpartum to determine glucose metabolism status and ensure consistent classification of glycemic outcomes. The Research Ethics Committees of Porto Alegre approved the protocol (CAE no. 00914312.0.1001.5327).

### Physical activity measurement

#### Accelerometry

Participants wore an accelerometer (wGT3x-BT, ActiGraph, USA) attached to the right side of the waist, secured by an elastic band, and aligned to the knee. They were instructed to use the device for seven days, during the 24 hours, except for water-based activities. The devices were activated in the Actilife software (v. 6.13.4, ActiGraph, USA) to record raw acceleration data at a frequency of 30 Hz across the three axes, starting at 2 pm on the day of the clinic visit and ending at the same time seven days later.

Data was processed using the GGIR package in the R software (v. 2.5-0, R project, New Zealand) (^14^). Post-collection autocalibration was performed, followed by a triaxial vector magnitude calculation for each 5-second epoch. We used the Euclidean norm minus one (Equation 1):

(1) $$\text{Vm =}\sqrt{{\text{x}}^{2}}+{\text{y}}^{2}+{\text{z}}^{2}-1\text{g}$$

rounding negative values to zero. Epochs were classified as MVPA when acceleration exceeded 69 mg (mg = 1 g × 10^-3^), corresponding to activities with an intensity greater than 3 metabolic equivalents (^15^). Ten-minute bouts of MVPA were identified by periods of sustained intense activity above the MVPA threshold, tolerating up to 20% of the duration at a lower intensity. Missing data were imputed using the mean acceleration intensity for the corresponding time of day on days with available data (^14^,^15^).

Data were considered valid if all of the following criteria were met: 1) data recorded for epoch within the 24-hour cycle in the sample, even combining different days; 2) calibration error below 0.02 g (1 g = 9.8 m/s²) following self-calibration; 3) at least four days with a minimum of 16 hours of wear time; and 4) at least one weekend day (Saturday or Sunday) (^16^). Aspects of data acquisition, including number of devices used, are described in Supplementary material.

#### Questionnaire

To provide contextual information for the physical activity estimates and to obtain self-reported data on physical activity, the international physical activity questionnaire (IPAQ) long-form was administered. Leisure time and commuting physical activity domains were used to estimate self-reported MVPA. The questionnaire assesses the frequency and duration of physical activities performed for 10 minutes or longer over the previous week. The original sample included Brazilian individuals (^17^). The proportion of women meeting the WHO physical activity recommendations for pregnancy and postpartum, defined as at least 150 minutes of MVPA per week, was estimated on the described estimates (^6^).

#### Measurement and definition of covariates

Certified research assistants collected sociodemographic and gestational data using questionnaires and reviewed medical records during pregnancy. Data collection began during a prenatal care visit and continued via telephone until participants visited the research center, where anthropometric measurements and complementary information were obtained. Information on weight gain and diseases during pregnancy (hypertension, preeclampsia, eclampsia, hemorrhage, infection) was retrieved from records in the prenatal booklet. The participant’s weight, height, and body mass index were recorded while barefoot and wearing light clothing, following standardized protocols. Body mass index was calculated as kg/m². Participants were grouped by age, family income, level of education, number of children, self-declared race/ethnicity (Caucasian and non-white), and body mass index classification.

#### Statistical analysis

Categorical variables were described as frequencies and percentages, while continuous variables were reported as means with standard deviations or medians with interquartile ranges. The data was evaluated using histograms and the Shapiro-Wilk test.

Given the right-skewed distribution of unbouted total weekly MVPA time, associations between participant characteristics and MVPA were evaluated through a generalized linear model using a gamma distribution with a log link. The average MVPA for each category of the explanatory variables was estimated by unadjusted models and adjusted models based on the marginal means of each variable included.

The statistical significance of the association between the adjusted models was investigated using the Wald test with a 5% significance level. A 10% significance level in unadjusted models to minimize confounding bias was used to guide variable inclusion. All analyses were conducted in R software (version 4.0.2, R project, New Zealand).

## RESULTS

**Figure 1:**
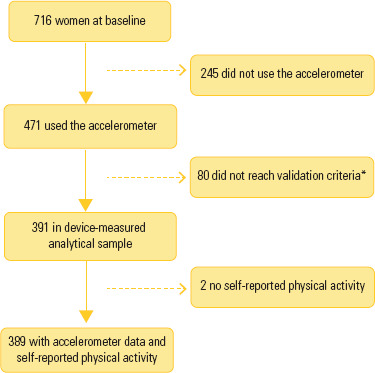
Sample flowchart. Self-reported physical activity based on the IPAQ. *With less than 16h/day of use and less than 4 days/week.

Of the 716 eligible participants, 245 were excluded due to non-use of the accelerometer or incomplete baseline visit. After further exclusion of 80 participants who did not meet wear time validity criteria, 391 women remained for analysis of device-measured physical activity data, and 389 had data available for self-reported PA (Figure 1). 

Participants were assessed at a median of 7.3 (interquartile range [IQR]: 4.0, 14.0) months postpartum, with a mean age of 33.8 years (±5.7; Table 1). 

**Table 1:** Participant’s characteristics of women with recent GDM

Variables	n*	Total sample (n = 716)	Accelerometer (n = 471)	Valid accelerometer data** (n = 391)
Age (y, [SD])	707	33.66 (5.86)	33.52 (5.87)	33.80 (5.69)
<30		165 (23.34%)	109 (23.49%)	87 (22.25%)
30-35		198 (28.34%)	134 (28.88%)	117 (29.92%)
>35		344 (48.66%)	221 (47.63%)	187 (47.83%)
Race/ethnicity	714			
Caucasian		293 (41.04%)	201 (42.86%)	169 (43.22%)
Non-white		421 (58.96%)	268 (57.14%)	220 (56.78%)
Level of education	716			
Primary		175 (24.44%)	114 (24.26%)	97 (24.81%)
High School		402 (56.15%)	261 (55.53%)	212 (54.22%)
University		139 (19.41%)	95 (20.21%)	82 (20.97%)
Family income (minimum wage†)	702			
≤1		125 (17.81%)	84 (18.10%)	68 (17.62%)
1-2		265 (37.75%)	174 (37.50%)	142 (36.79%)
2-3		167 (23.79%)	112 (24.14%)	95 (24.61%)
>3		145 (20.6%)	94 (20.26%)	81 (20.98%)
Living with partner	716	633 (88.41%)	415 (88.11%)	340 (87.18%)
Number of children	571			
<2		477 (83.54%)	308 (83.02%)	254 (81.94%)
3		51 (8.93%)	35 (9.43%)	33 (10.65%)
>3 or more		343 (7.53%)	28 (7.55%)	23 (7.42%)
Time after delivery (months)#	698	8.00 [4.37; 14.17]	7.43 [4.03; 14.03]	7.32 [3.98; 14.02]
Weight gain during pregnancy (mean)	703	8.21 (7.23)	8.04 (7.5)	8,37 (7.0)
Postpartum BMI – post-pregnancy	708	30.67 (4.91)	30.52 (4.75)	30.42 (4.63)
Postpartum BMI classification‡	710			
<30 kg/m^2^		313 (44.08%)	210 (44.97%)	176 (45.01%)
≥30 kg/m^2^		397 (55.92%)	257 (55.03%)	215 (54.99%)
OGTT results	716			
Standard		223 (31.2%)		125 (32%)
Prediabetes		378 (52.8%)		220 (56%)
Diabetes		101 (14,1%)		41 (10.5%)
Missing		14 (2%)		5 (1.3%)
Comorbidity during pregnancy^§^ (%)	682	354 (51.91)	229 (49.67)	187 (48.45)

BMI: body mass index; OGTT: oral glucose tolerance test.

* Numbers vary due to missing data

** at least four days with a minimum of 16 hours of wear time, and at least one weekend day. For records shorter than 7 days, random sampling of clusters of 1-6 days were performed for each participant with 7 days of use.

# Median and interquartile range.

† Based on the 2024 Brazilian minimum wage (BRL 1320.00 ≈ USD 233.00).

‡ WHO (2020).

§ Hypertension, preeclampsia, eclampsia, hemorrhage, and infection.

The sample was predominantly non-white (56.8%), had not a university degree (79.0%), and reported an income ≤2 minimum wages (54.4%). The mean body mass index was 30.42 kg/m² (SD 4.63), and 49.67% of participants had at least one additional comorbidity during the most recent pregnancy. Only 12.8% were not living with a partner. These characteristics were consistent between those with valid accelerometer data and the overall sample. 

Most participants delivered via cesarean section (62.4%) and had more than one previous pregnancy (79.5%). The most frequently reported gestational comorbidities included hypertension, preeclampsia, eclampsia, hemorrhage, and infection. Postpartum oral glucose tolerance test results were available for 391 participants: 125 (32.0%) had standard glucose tolerance, 220 (56.3%) had prediabetes, and 41 (10.5%) met diagnostic criteria for diabetes. Data were missing for 5 participants (1.3%).

**Figure 2:**
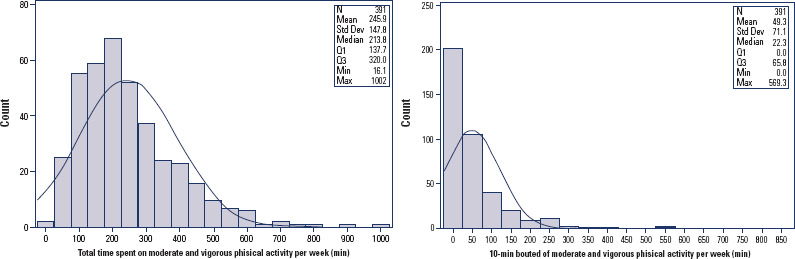
Frequency distributions of the 7-day mean of time spent on moderate or vigorous physical activity. (A) Total; (B) 10-minute bouts.

The distribution of weekly MVPA measured by accelerometer was right-skewed, presenting a mean of 245.86 minutes (SD 147.8) and a median of 213.8 minutes (IQR: 137.74-320.0). A similar pattern was observed when analyzing only 10-minute bout activities, with a mean of 49.3 minutes (SD 71.1 minutes) and a median of 22.3 minutes (IQR: 0-65.8). This wide variability indicates that most physical activity was performed in short incidental bursts rather than sustained periods (Figure 2).

**Figure 3:**
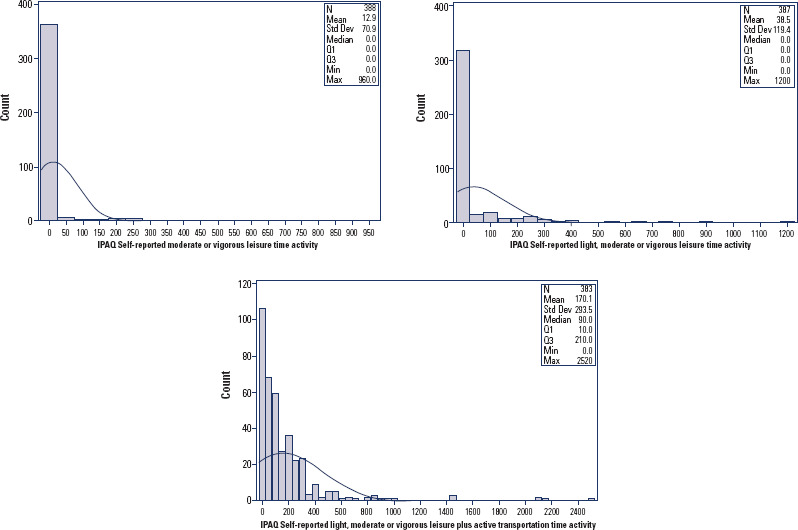
Frequency distributions of the 7-day physical activity reported from the IPAQ. (A) Only leisure-time moderate/vigorous physical activity; (B) Leisure time walking, moderate-to-vigorous activity; (C) Commuting-time walking, moderate or vigorous physical activity.

Self-reported time spent in MVPA was also right-skewed across all measures. For total self-reported MVPA per week, the mean was 12.9 minutes (SD 70.9), and the median was 0 (IQR: 0-0). Levels increased when walking was included, especially when walking was counted (Figure 3).

The distribution of weekly minutes spent in MVPA according to participants’ sociodemographic and perinatal characteristics is summarized in Table 2. Adjusted means are presented to control for potential confounding variables. 

**Table 2:** Weekly accelerometer-measured moderate-to-vigorous physical activity in women with recent gestational diabetes by participant characteristics

Variables	Unadjusted mean# (95% CI)	p**	Adjusted mean*# (n = 385; 95% CI)	p**
Age group		0.269		
<30	263.20 (232.47-298.00)			
30-35	230.24 (206.87-256.26)			
>35	247.57 (227.47-269.45)			
Race/ethnicity (n = 390)		0.0584		0.2191
Caucasian	230.09 (238.61-278.69)		257.46 (227.68-291.13)	
Non-white	257.88 (210.50-251.50)		277.51 (250.52-307.41)	
Living with partner		0.0532		0.0702
Yes	239.81 (225.22-255.34)		246.25 (229.06-264.73)	
No	285.07 (242.03-335.75)		290.14 (244.75-343.95)	
Level of education (n = 391)		0.0219		0.0448
Primary	276.23 (245.85-310.37)		286.71 (250.05-328.75)	
High school/university	235.85 (220.49-252.27)		249.20 (226.54-274.11)	
Family income (minimum wage†)		0.269		
≤1	278.02 (241.53-320.03)			
1-2	241.85 (219.41-266.58)			
2-3	242.65 (215.42-273.32)			
>3	231.15 (203.19-262.96)			
Postpartum BMI classification‡ (n = 391)		0.2141		
<30	256.00 (234.58-279.38)			
≥30	237.57 (219.51-257.12)			
Comorbidity during pregnancy (n = 386)		0.0279		0.0225
Yes	229.60 (210.98-249.86)		249.70 (223.38-279.13)	
No	262.02 (241.40-284.41)		286.13 (255.46-320.49)	
Number of children (n = 309)		0.2732		
<2	242.88 (226.06-260.95)			
3	288.83 (263.50-352.74)			
>3	243.34 (190.50-310.85)			

BMI: body mass index.

* Marginal mean is obtained through a generalized linear model with gamma distribution and log as link a function.

** *p* = Wald’s test.

# minutes/week.

† Based on the 2024 Brazilian minimum wage (BRL 1320.00 ≈ USD 233.00.

‡ WHO (2020). Variables included in models were race/ethnicity, living with a partner, level of education, and comorbidity during pregnancy.

Variation was observed across several characteristics. Notably, race/ethnicity, living with a partner, level of education, and comorbidity during pregnancy were associated with total MVPA (device-measured) in unadjusted models at the 10% significance level. After adjusting for age, only primary level of education and absence of comorbidities during pregnancy remained independently associated with total time in MVPA. Additionally, living with a partner was linked to lower levels of device-measured MVPA. No other associations were identified in the sample.

Based on total accelerometer data, adherence to current MVPA guidelines during pregnancy and postpartum was 71.6% (95% CI: 66.9-76.0). However, it reduced to 8.4% (95% CI: 5.9-11.6) when considering only 10-minute bouts, similar to rates obtained from self-reported leisure-time activities. When self-reported time also included commuting, 26.8% (95% CI: 22.5-31.5) of the sample met the recommendations. Across all measurements, participants with only a primary level of education demonstrated higher physical activity levels (Table 3).

**Table 3:** Description of weekly moderate-to-vigorous physical activity in women with recent GDM

Variables	Accelerometer (n = 391)		IPAQ (n = 389)	
	Total MVPA**	10-minutes bout MVPA**	Leisure total**	Leisure and commuting total**
Mean (SD):	245.86 (147.78)	49.25 (71.11)	38.49 (119.35)	170.09 (293.46)
Median (q1; q3)	213.83 (137.74; 320.03)	22.31 (0; 65.84)	0 (0; 0)	90 (10.00; 210.00)
Active* (%; 95% CI)	71.61 (66.86; 76.03)	8.44 (5.88; 11.65)	7.42 (5.02; 10.48)	26.85 (22.52; 31.54)
Primary#	n = 97		n = 96	
Mean (SD)	276.23 (155.89)	57.00 (67.43)	53.14 (164.84)	194.67 (288.92)
Median (q1; q3)	256.59 (157.28; 345.70)	31.14 (11.00; 74.83)	0 (0; 0)	110.00 (60.00; 225.00)
Active* (%; 95% CI)	76.29 (66.58; 84.34)	11.34 (5.80; 19.39)	8.25 (3.63; 15.61)	26.80 (18.32; 36.76)
High school/university#	n = 294		n = 293	
Mean (SD)	235.85 (143.87)	46.70 (72.21)	33.79 (100.42)	162.32 (294.95)
Median (q1; q3)	207.12 (131.25; 306.76)	19.51 (0; 62.05)	0 (0; 0)	75.00 (0; 200.0)
Active* (%; 95% CI)	70.07(64.48; 75.25)	7.48(4.75; 11.11)	7.14 (4.45; 10.71)	26.87 (21.89; 32.32)

MVPA: moderate-to-vigorous physical activity (acceleration > 69 mg); IPAQ: international physical activity questionnaire; SD: standard deviation; 95% CI: 95% confidence interval; Total: walking, MVPA.

* At least 150 minutes/week of MVPA (WHO, 2020).

** Minutes/week.

# Level of education.

## DISCUSSION

Women with GDM within two years postpartum who used a waist accelerometer for seven days spent 245.9 (±147.8) minutes/week of MVPA. When considering only 10-minute bouts, the mean decreased to 49.2 (SD 71.11) minutes/week. Although 71.6% (95% CI: 66.7-76.0) met the 150 minutes/week MVPA target, only 8.4% (95% CI: 5.9-11.6) achieved it in 10-minute bouts. Higher education was associated with lower activity levels. Similarly, pregnancy comorbidities also corresponded to reduced physical activity. Based on self-reported activities lasting 10 minutes, the mean time spent on MVPA at leisure was 38.5 (±119.3) minutes/week, increasing to 170.0 (±293.5) minutes/week when commuting time was included, with 7.4% (95% CI: 5.0-10.5) and 26.8 (95% CI: 22.5-31.5) meeting the guideline, respectively. 

This study appears to be the first on device-measured MVPA in postpartum women with recent GDM. Therefore, comparisons of our results with previously published studies about the topic must account for the differences in the peripartum time, women’s age, and methodological approaches for accelerometer protocols and data analysis. A Canadian study of 109 women aged 36.3 years at 2.9 years after birth reported a similar pattern but lower levels of device-measured MVPA: women engaged in MVPA an average of 136 minutes/week, with 31% meeting activity recommendations. On 10-minute bouts, the mean was reduced to 7 minutes/week of MVPA, and only 7% were physically active (^18^). In another study in Norway involving 23 women with GDM aged 36.6 years one year after birth, 35% of the sample was physically active, with an average device-measured moderate PA of 254 minutes/week (^9^). Of note is that both studies used Actigraph’s algorithm to define intensity, while this study’s estimations originated from raw data. The analysis comprised 5-second epochs, and the other studies used 1-minute epochs. Since longer epochs tend to dilute short bursts of activity, this is more important in older individuals (^19^). This methodological difference is notable, potentially capturing shorter bursts of habitual activity, as extended epochs underestimate lower PA at lower acceleration ranges due to a dilution effect. Furthermore, a study conducted in Finland found an average of moderate PA of 350 minutes/ week in slightly older women with recent GDM, 4-6 years postpartum (^20^).

Studies using the IPAQ have demonstrated generally low postpartum physical activity levels (^21^,^22^). However, by including leisure activities and commuting, this study identified a higher proportion of women meeting the recommended physical activity targets. The low prevalence of physically active women for 10-minute bouts of MVPA has been well documented, including the postpartum period (7-9,18). In adults, removing the requirement for 10-minute bout activities doubles the proportion of meeting the PA recommendations (^23^). In fact, the WHO has dropped the 10-minute bouts requirement in their most recent guidelines, as total MVPA is associated with a range of important health outcomes applicable to any bout duration (^24^).

Defining 150 minutes/week to classify physically active women based on device-measured PA warrants further discussion. First, WHO has maintained this threshold for postpartum women, regardless of the method used, while adding a second tier of 300 minutes/week for active individuals (^6^). Second, a large meta-analysis using harmonized individual data from accelerometer-based PA showed an inverse association between mortality and increasing levels of accelerometer-measured total MVPA, with a plateau of approximately 140 minutes/week (^25^). Therefore, the 150-minute threshold seems adequate for device-measured PA for women at postpartum.

The observed association between lower levels of education and higher levels of PA differs from other studies in high-income countries, where women with higher socioeconomic status are more active postpartum (^26^,^27^). Higher levels of GPA among less socially privileged women reflect their work duties captured through the accelerometer. 

The limitations of this study comprised the sample: women were recruited from health services in selected Brazilian cities and do not represent all Brazilian women with GDM at postpartum; not all women selected completed the accelerometer protocol, potentially introducing selection bias. However, the characteristics of those with valid accelerometer information were similar to those of the whole sample. Lastly, self-reported PA assessed by IPAQ only included two dimensions, leisure and commuting, excluding occupational physical activity and consequently underestimating total MVPA. 

The robustness of this study is supported by data collection following protocols designed priori and with highly standardized procedures performed by certified personnel. Additionally, PA was assessed using raw accelerometry data rather than basing the findings on fixed algorithms provided by the device, which enabled greater control at each step of the data processing and enhanced comparability with other extensive studies.

In conclusion, a substantial proportion of women with GDM enrolled on average about seven months postpartum was physically active when total device-based MVPA was considered. However, when applying the 10-minute bout activities, a large fraction would be classified as inactive. Women with lower-levels of education had higher total levels of PA, which may reflect their work/commuting daily duties not captured when only considering 10-minute bouts PA. These findings provide crucial information for public policies to encourage and support the practice of feasible PA at leisure in the postpartum period.

## References

[1] GBD 2021 Diabetes Collaborators (2023). Global, regional, and national burden of diabetes from 1990 to
2021, with projections of prevalence to 2050: a systematic analysis for the
Global Burden of Disease Study 2021. Lancet..

[2] IDF Diabetes Atlas 2021 IDF Diabetes Atlas [Internet].

[3] Goveia P, Cañon-Montañez W, Santos DP, Lopes GW, Ma RCW, Duncan BB (2018). Lifestyle Intervention for the Prevention of Diabetes in Women
with Previous Gestational Diabetes Mellitus: A Systematic Review and
Meta-Analysis. Front Endocrinol (Lausanne).

[4] Li N, Yang Y, Cui D, Li C, Ma RCW, Li J (2021). Effects of lifestyle intervention on long-term risk of diabetes
in women with prior gestational diabetes: A systematic review and
meta-analysis of randomized controlled trials. Obes Rev..

[5] Retnakaran M, Viana LV, Kramer CK (2023). Lifestyle intervention for the prevention of type 2 diabetes in
women with prior gestational diabetes: A systematic review and
meta-analysis. Diabetes Obes Metab..

[6] World Health Organization (2020). WHO guidelines on physical activity and sedentary behaviour.

[7] Hesketh KR, Evenson KR, Stroo M, Clancy SM, Østbye T, Benjamin-Neelon SE (2018). Physical activity and sedentary behavior during pregnancy and
postpartum, measured using hip and wrist-worn accelerometers. Prev Med Rep..

[8] Wolpern AE, Bardsley TR, Brusseau TA, Byun W, Egger MJ, Nygaard IE (2021). Physical activity in the early postpartum period in primiparous
women. J Sci Med Sport..

[9] Ziesler CPØ, Staff AC, Sugulle M, Moe K (2021). Low physical activity levels 1 year after pregnancy
complications. Pregnancy Hypertens.

[10] Ziesler CPØ, AC, Sugulle M, Moe K (2023). Corrigendum to “Low physical activity levels 1 year after
pregnancy complications” [Pregnancy Hyperten. 25 (2021)
136-142]. Pregnancy Hypertens.

[11] Engberg E, Stach-Lempinen B, Rönö K, Kautiainen H, Eriksson JG, Koivusalo SB (2018). A randomized lifestyle intervention preventing gestational
diabetes: effects on self-rated health from pregnancy to
postpartum. J Psychosom Obstet Gynaecol.

[12] Hod M, Kapur A, Sacks DA, Hadar E, Agarwal M, Di Renzo GC (2015). The International Federation of Gynecology and Obstetrics (FIGO)
Initiative on gestational diabetes mellitus: A pragmatic guide for
diagnosis, management, and care. Int J Gynaecol Obstet.

[13] Tornquist L, Tornquist D, Mielke GI, da Silveira MF, Hallal PC, Domingues MR (2023). Maternal Physical Activity Patterns in the 2015 Pelotas Birth
Cohort: From Preconception to Postpartum. J Phys Act Health.

[14] van Hees VT, Gorzelniak L, Dean León EC, Eder M, Pias M, Taherian S (2013). Separating movement and gravity components in an acceleration
signal and implications for the assessment of human daily physical
activity. PLoS One..

[15] Hildebrand M, VAN Hees VT, Hansen BH, Ekelund U (2014). Age group comparability of raw accelerometer output from wrist-
and hip-worn monitors. Med Sci Sports Exerc..

[16] de Paula D, Crochemore-Silva I, Griep RH, Duncan BB, Schmidt MI (2023). Accelerometry Measured Movement Behaviors in Middle-Aged and
Older Adults: Cross-Sectional Analysis of the ELSA-Brasil
Study. J Phys Act Health..

[17] IPAQ Questionário Internacional de Atividade Física –
Versão Longa [Internet].

[18] Gingras V, Vigneault J, Weisnagel SJ, Tchernof A, Robitaille J (2013). Accelerometry-measured physical activity and inflammation after
gestational diabetes. Med Sci Sports Exerc..

[19] Ayabe M, Kumahara H, Morimura K, Tanaka H (2013). Epoch length and the physical activity bout analysis: An
accelerometry research issue. BMC Res Notes.

[20] Sahrakorpi N, Engberg E, Stach-Lempinen B, Tammelin TH, Kulmala J, Roine RP (2022). Physical activity and health-related quality of life among
high-risk women for type 2 diabetes in the early years after
pregnancy. BMC Womens Health..

[21] Tsironikos GI, Potamianos P, Zakynthinos GE, Tsolaki V, Tatsioni A, Bargiota A (2023). Effectiveness of Lifestyle Interventions during Pregnancy on
Preventing Gestational Diabetes Mellitus in High-Risk Women: A Systematic
Review and Meta-Analyses of Published RCTs. J Clin Med..

[22] Bulguroglu HI, Bulguroglu M, Gevrek PTC (2023). Investigation of the effects of physical activity level on
functionality level and quality of life in the postpartum
period. J Health Popul Nutr..

[23] Prince SA, Roberts KC, Lang JJ, Butler GP, Colley RC (2022). The influence of removing the 10-minute bout requirement on the
demographic, behaviour and health profiles of Canadian adults who meet the
physical activity recommendations.

[24] Jakicic JM, Kraus WE, Powell KE, Campbell WW, Janz KF, Troiano RP (2019). Association between Bout Duration of Physical Activity and
Health: Systematic Review. Med Sci Sports Exerc..

[25] Ekelund U, Tarp J, Steene-Johannessen J, Hansen BH, Jefferis B, Fagerland MW (2019). Dose-response associations between accelerometry measured
physical activity and sedentary time and all cause mortality: systematic
review and harmonised meta-analysis. BMJ..

[26] Lynch KE, Landsbaugh JR, Whitcomb BW, Pekow P, Markenson G, Chasan-Taber L (2012). Physical Activity of Pregnant Hispanic Women. Am J Prev Med..

[27] Kracht CL, Drews KL, Flanagan EW, Keadle SK, Gallagher D, Van Horn L (2024). Maternal 24-h movement patterns across pregnancy and postpartum:
The LIFE-Moms consortium. Prev Med Rep.

[28] Koo TK, Li MY (2016). A Guideline of Selecting and Reporting Intraclass Correlation
Coefficients for Reliability Research. J Chiropr Med..

[29] da Silva SG, Evenson KR, Ekelund U, da Silva ICM, Domingues MR, da Silva BGC (2019). How many days are needed to estimate wrist-worn
accelerometry-assessed physical activity during the second trimester in
pregnancy?. PLoS One.

[30] Ricardo LIC, Wendt A, Galliano LM, de Andrade Muller W, Niño Cruz GI, Wehrmeister F (2020). Number of days required to estimate physical activity constructs
objectively measured in different age groups: Findings from three Brazilian
(Pelotas) population-based birth cohorts. PLoS One.

